# Educating Internal Medicine Residents About Breast Health Using a Jigsaw Teaching Strategy: An Interactive Curriculum Module

**DOI:** 10.15766/mep_2374-8265.11652

**Published:** 2026-11-12

**Authors:** Rebecca Zeidman, Anne Chen, Yashvi Patel, Rosemarie L. Conigliaro, Katherine Killian, Sneha Shrivastava

**Affiliations:** 1 Fellow in Endocrinology, Department of Medicine, Donald and Barbara Zucker School of Medicine at Hofstra/Northwell; 2 Assistant Professor, Department of Medicine, Donald and Barbara Zucker School of Medicine at Hofstra/Northwell; 3 Associate Program Director, Internal Medicine Residency, Atlantic Health Overlook Medical Center; 4 Professor of Medicine Emeritus, Donald and Barbara Zucker School of Medicine at Hofstra/Northwell; 5 Associate Professor, Department of Medicine, Donald and Barbara Zucker School of Medicine at Hofstra/Northwell; Program Director, Internal Medicine Residency, Lenox Hill Hospital; 6 Associate Professor, Department of Medicine, Renaissance School of Medicine at SUNY Stony Brook

**Keywords:** Women's Health, Breast Health, Internal Medicine, Genetic Counseling, Breast Cancer Screening

## Abstract

**Introduction:**

Education in women's health is lacking in internal medicine (IM) residency training despite women making up half the population. With increasing recognition of women's unique health care needs and recent guideline updates for breast cancer (BC) screening, we utilized an interactive teaching strategy, the Jigsaw Teaching Strategy (JTS), to increase resident comfort with BC screening at 2 large New York (NY) IM residency programs.

**Methods:**

One hundred eighty-six IM residents across 2 residency programs participated in a one-hour JTS session in 2024. JTS is a cooperative teaching technique that assigns each learner to become an “expert” on one topic and then brings learners together in mixed groups to teach their individual topics to one another. Four cases were developed to cover BC guidelines and BC risk assessment across all ages. Resident pre- and postsession surveys were conducted to assess residents’ comfort and knowledge of this topic.

**Results:**

Response rates for the pre- and postsession survey were 52% (96/186) and 38% (72/186), respectively. Residents’ comfort level with breast imaging improved (presession mean = 2.86 [*SD* = 0.82], postsession mean = 4.01 [*SD* = 0.72], *P* < .001). Resident comfort with performing BC risk stratification improved (presession mean = 2.44 (*SD* = 0.81), postsession mean = 3.99 (*SD* = 0.70), *P* < .001). Post session, 98% of the residents correctly identified the recommended age(s) to begin screening, and 98% correctly identified first-line imaging modality.

**Discussion:**

The implementation of JTS was feasible and well received by residents, improving their self-reported comfort with BC screening and risk assessment.

## Educational Objectives

By the end of this session, learners will be able to:

1. Stratify patients who are at high risk for breast and other related cancers.
2. Describe the breast cancer screening guideline recommendations from national bodies.
3. Interpret mammogram reports.
4. Discuss risk versus benefits of breast cancer screening in older patients.
5. Discuss when to stop screening for breast cancer.

## Introduction

Women's preventative health is a crucial component of comprehensive medical care, and it has historically been underrepresented in medical training. As the recognition for women's unique health care needs increases, primary care providers must be equipped with the knowledge and tools to deliver high-quality care to women.^1^ Previous research has highlighted significant gaps in internal medicine (IM) residency training in women's health, with limited integration of these topics into IM curricula.^2,3^

Despite the recognized need for improved women's health training in IM residency, published curricula on breast health remain limited. Current programs rely on traditional lecture-based teaching or small-group case-based sessions.^4^ Prior research has shown that Women's Health Tracks (WHTs) and problem-based learning (PBL) workshops are effective at improving residents’ knowledge, comfort, and clinical exposure to critical women's health topics.^5^

Among the many women's health–related topics, breast cancer (BC) screening and risk assessment are among the most relevant and essential to the education of IM residents. They are particularly complex domains that require integration of multiple, often conflicting guidelines from various organizations. Clinicians must engage in shared decision making with patients, weighing individualized risk factors and interpreting risk assessments. These characteristics make breast health topics well suited for peer-teaching approaches. Learning to synthesize information and explaining to peers mirror the discussions regarding screening with patients. At our institutions, breast health topics were previously taught through traditional didactics during ambulatory medicine curriculum. However, given the complexity of breast cancer screening guidelines, the need for residents to synthesize and apply them across various clinical scenarios calls for a more interactive approach.

Jigsaw Teaching Strategy (JTS) is a cooperative teaching technique that assigns each learner to become an “expert” on one topic and then brings learners together in mixed groups to teach their topics to one another. As an interactive and cooperative learning strategy, it has been used to improve engagement, communication skills, and interpersonal relationships, with both students and faculty deeming it effective.^6,7^ The jigsaw strategy is primarily rooted in cooperative learning, constructivism, and social interdependence theory. JTS sessions have demonstrated increased knowledge and self-confidence regarding transgender care among medical students.^8^ More recently, a JTS workshop among IM residents improved the diagnosis and management of geriatric women's health conditions, including menopause, osteoporosis, urinary incontinence, and abnormal uterine bleeding.^9^ The workshop significantly increased residents’ knowledge and confidence, with residents expressing high satisfaction and requesting further women's health education.

The goal of this curriculum is to apply the JTS to increase confidence in breast health among IM residents. Our innovation expands on previous works by implementing the JTS in a novel topic, in a shorter, one-hour format, and across both virtual and in-person formats, thereby broadening the approaches’ generalizability and efficiency for core curriculum topics. By comparing residents’ comfort in breast health topics before and after the intervention, we anticipate that this teaching method will improve residents’ confidence in applying these principles to patient care.

## Methods

### Activity Design and Participants

Participants were IM residents, postgraduate years (PGY) 1 to 3, at 2 residency programs within the same large health system in New York. We implemented sessions during ambulatory blocks in the fall of 2024. One program offered all sessions virtually, while the other offered all sessions in person. Faculty facilitators with clinical experience in BC screening and risk assessment were present for each session (Appendix A).

Prior to implementation, foundational knowledge related to BC screening was covered at both sites as part of the existing ambulatory medicine curriculum. The facilitators selected for these sessions required no additional formal training other than a thorough review of the facilitator guide (Appendix A). This curriculum was deemed exempt as determined by Northwell's institutional review board.

### Curriculum Development

We created 4 case-based scenarios on breast health: (1) identifying first-line breast imaging for a young woman with symptoms, (2) stratifying high-risk patients for BC screening, (3) reviewing BC screening guidelines, and (4) discussing discontinuation of BC screening when appropriate (Appendix C). We created facilitator and participant versions of the cases to give to the facilitators and residents, respectively, prior to the session (Appendices B and C). We created a PowerPoint slide deck containing the overall goals, learning objectives, and a summary of the major take-home points (Appendix D). Each resident had 2 group assignments: one group where they would become an expert about their case-based scenario, and another group to which they were expected to teach others about their case.

### Curriculum Evaluation

We created pre- and posttest surveys to be administered prior to and immediately after the session (Appendix E). We developed the survey items based on the learning objectives derived from the 4 case-based scenarios, and they were reviewed by content experts with clinical and educational expertise in women's health. We created knowledge multiple-choice questions to assess core concepts based on BC screening and risk assessment tools (Kirkpatrick Level 2), and the 5-point Likert scale items were adapted to measure learner satisfaction and comfort level (5 = *very satisfied or very comfortable*; Kirkpatrick Level 1; Appendix E).^10^ The survey instruments were not formally validated using psychometric methods. The survey data were collected anonymously via Google Forms. Since the survey data were collected anonymously and separately, individual pre- and postsession responses could not be linked.

### Implementation of Session

The session began with facilitators sharing the presession survey, PowerPoint slides with goals, learning objectives, and, JTS strategy along with the group assignments for the residents and facilitators (Appendices E and D). The introductory PowerPoint presentation took 10 minutes. In-person participants would physically move to the table with their groupmates, while virtual participants were assigned breakout rooms. The size of each small group was dependent on how many residents were present in that session, but they were divided into a total of 4 groups, with each group assigned one of the 4 scenarios. Each small group ranged from approximately 4 to 8 residents depending on the total number of residents present at the session. At each breakout group or room, there was at least one facilitator. In each group, the residents discussed the case and the answers for their scenario, for which they were allowed to use the internet and access guidelines to help them.

The facilitator guide contained guidelines and best practices for facilitators to facilitate the small-group discussion to cover all the learning points for their scenario. The first round of small groups was allotted 5 minutes to discuss their assigned scenario. Subsequently, all the residents were rearranged into their second group assignment, where each new group now included residents from each of the 4 scenario groups. With this new group assignment, residents discussed all 4 cases since each resident was an “expert” in their respective scenarios. The second round of small groups was allotted 20 minutes to discuss the 4 cases. Afterwards, all residents reconvened into one unified group, and the facilitators used the PowerPoint to debrief and highlight the main learning points from each scenario, estimated to take 15 minutes to present. The total session was one hour.

### Statistical Analysis

Since pre- and postsession surveys were collected anonymously and could not be linked to individual responses, an unpaired *t*-test was used to compare pre- and postsession means on Likert scale questions on comfort (1 = *very uncomfortable*; 5 = *very comfortable*). Descriptive statistics were used to analyze demographic data, knowledge questions, satisfaction items, and modality preference. Statistical analysis was performed using Microsoft Excel software. A *P*-value of < .05 was considered statistically significant.

## Results

Of 186 residents, 96 responded to the presession survey and 72 to the postsession survey (response rates of 51.6% and 38.7%, respectively; Table). Of the presession survey respondents, 43% were PGY 1, 30% PGY 2, and 27% PGY 3. Forty-nine percent identified as female.

**Table.**
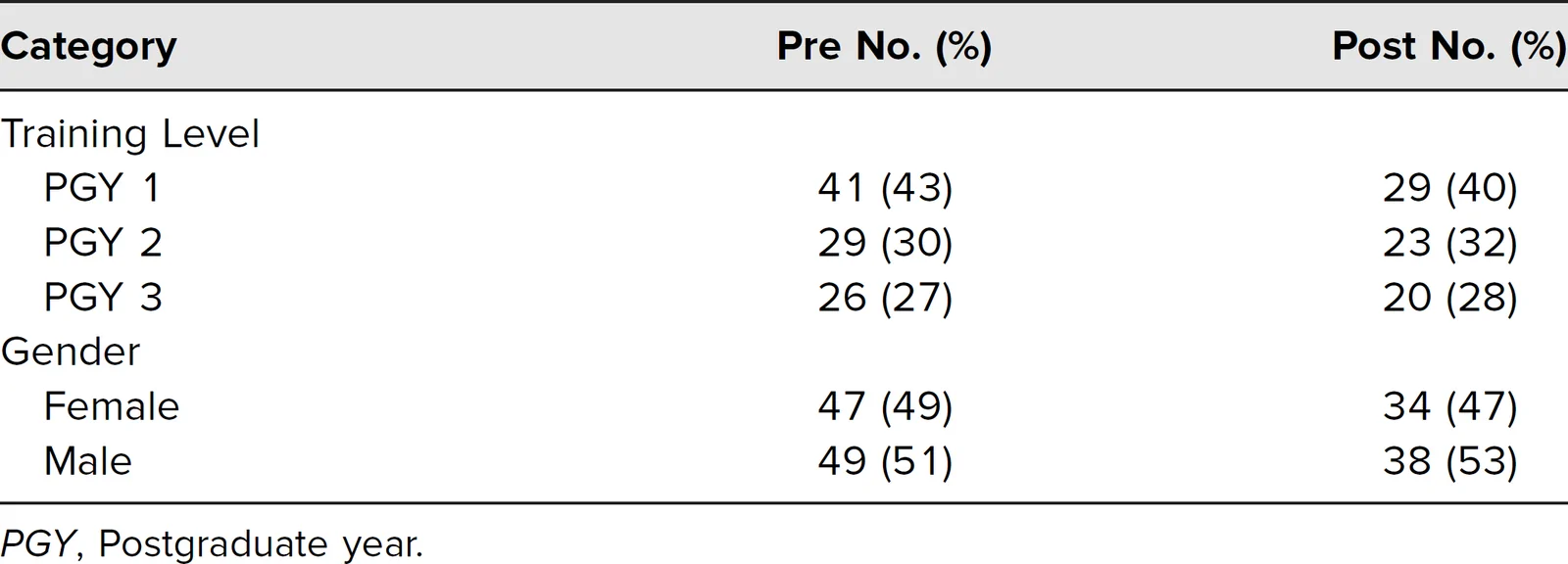
Resident Demographics for Pre- (*N* = 96) and Postsession (*N* = 72) Surveys

IM residents’ comfort level with breast imaging improved significantly after the session (presession *M* = 2.86 [*SD* = 0.82], postsession *M* = 4.01 [*SD* = 0.72], *P* < .001; Figure). Residents were significantly more comfortable performing risk stratification assessments after the session (presession *M* = 2.44 [*SD* = 0.81], postsession *M* = 3.99 [*SD* = 0.70], *P* < .001). On the postsession knowledge survey, 98% of the residents correctly identified the recommended age(s) to begin screening, and 98% correctly identified the first-line imaging modalities. Since pre- and posttest session knowledge questions were not identical and surveys were not linked to individual respondents, direct pre-to-post comparison of knowledge scores was not performed.

**Figure.**
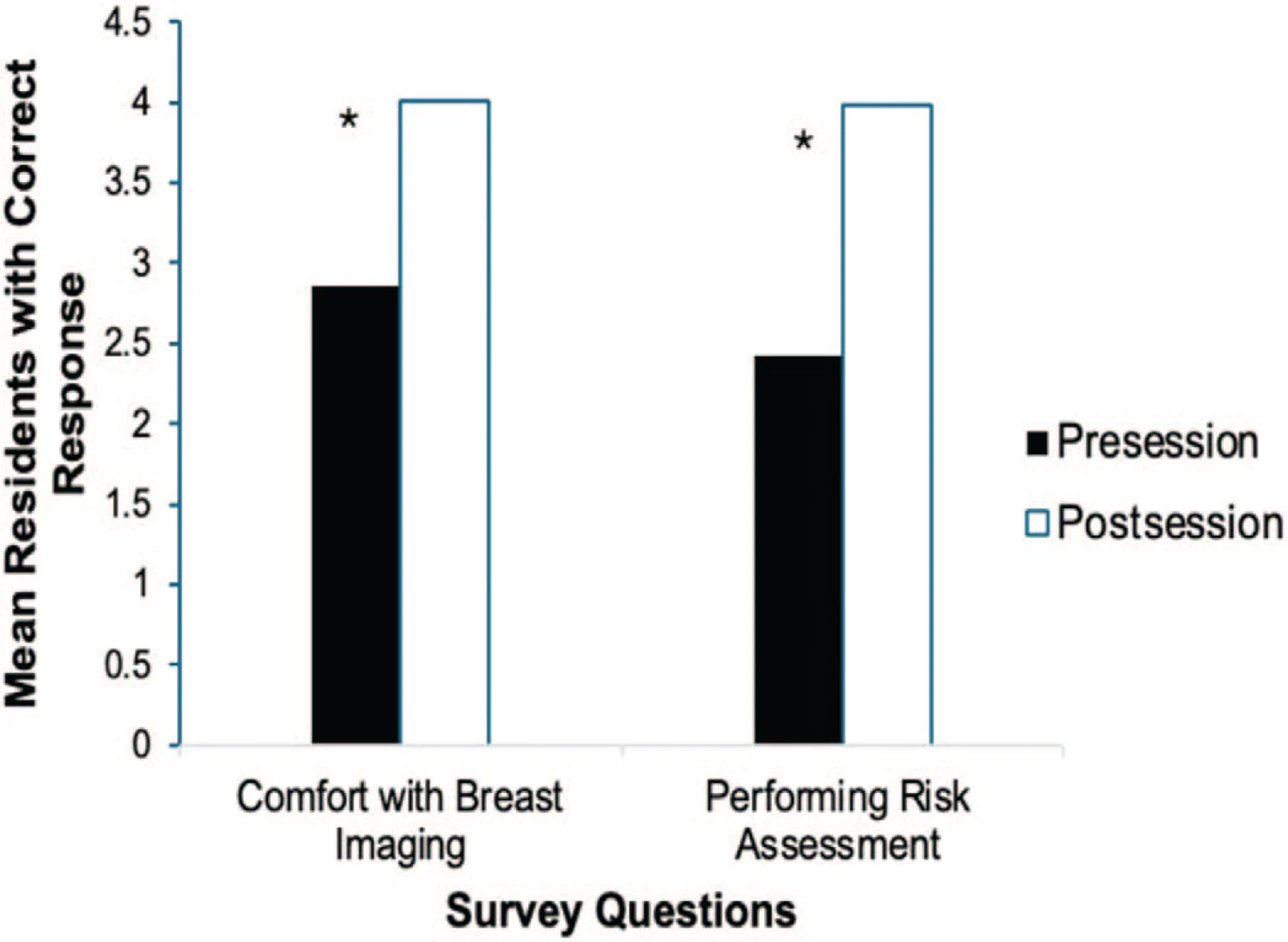
Mean resident comfort before (*N* = 96) and after (*N* = 72) the Jigsaw Teaching Strategy session on a 5-point Likert scale (1 = *very uncomfortable*; 5 = *very comfortable*). Asterisk designates significance at *P* < .001.

Among the residents who participated virtually, 4% preferred in person, 88% preferred virtual, and 8% had no preference. Of 45 residents in person, 40% preferred in person, 42% preferred virtual, and 17% had no preference.

## Discussion

We developed a case-based curriculum utilizing JTS, a peer-led teaching approach to foster self-directed learning. Our educational innovation allowed residents to move beyond lecture-based learning and engage in active learning regarding breast health topics. Our results demonstrate that a breast health curriculum utilizing JTS was feasible, well received, and associated with increased resident self-reported comfort in BC screening and risk assessment. On the immediate postsession survey, residents demonstrated knowledge of key breast health concepts. However, given the limitations of the knowledge assessment and the unlinked survey design, these findings primarily support the feasibility of JTS for delivering breast health content in different learning environments, in person and virtual.

In implementing the JTS model, we realized that the success of the session did hinge on resident participation and engagement. A key lesson learned during development was the need for high facilitator-to-group ratios in the virtual setting. We observed that in person, it was easier to have fewer facilitators to encourage discussion. However, when completed virtually, engagement was improved when there was a facilitator for each breakout room. Halfway through implementing the JTS sessions, we realized that residents in the in-person sessions often left prior to completing the postsession surveys. To counter this, we rearranged the slides to emphasize filling out the postsession survey before residents logged off, although this effort yielded mild success. Ultimately, our results reflect that the JTS model, whether implemented virtually or in person, led residents to feel more comfortable with breast health topics. These outcomes provide evidence that JTS can be adapted effectively across different modalities, which is crucial for curriculum developers when managing asynchronous learners.

Our study did find improvements in self-reported comfort in both virtual and in-person groups, highlighting the flexibility of JTS across different learning environments. While residents preferred the virtual format, they also viewed the in-person format favorably. These findings are encouraging for curriculum developers who must accommodate all learners. It is important to note that residents were exposed to only one modality and did not have direct comparison evidence. These preferences are, therefore, interpreted with caution. Self-reported comfort improvements were observed regardless of session modality. The major strength of our curriculum was ease of implementation of JTS in an in-person and virtual format that required minimal resources for a core women's health topic of breast cancer screening.

### Limitations

First, our study utilized a post-intervention knowledge assessment using 3 multiple-choice questions, which may not fully represent residents’ ability to apply clinical reasoning and limits the depth of our cognitive assessment. Because the knowledge questions were not validated or identical between the pre- and postsession surveys, these findings should be interpreted cautiously and only be used to describe resident knowledge after the JTS session. Additionally, the results cannot be used to compare the efficacy of JTS versus traditional teaching models, as residents were exposed to only the JTS model for this topic. Furthermore, our primary outcomes were self-reported, which is susceptible to response bias and may reflect an overestimation of residents’ abilities. The survey instruments we used in this study were not formally validated using psychometric methods; therefore, findings related to comfort and satisfaction should also be interpreted with caution.

Additionally, the response rates were also relatively low, which introduces the possibility of nonresponse bias. The difference in attrition rates between the virtual and in-person groups also presents a selection bias, as residents who completed the virtual sessions may have logged off prematurely before completing the postsession survey. Regarding modality preference, residents were exposed to only one format and did not experience both; therefore, preferences for virtual versus in-person delivery should be interpreted cautiously and also do not reflect comparison of the 2 modalities.

Furthermore, the sessions were implemented at 2 residency programs within a single health system, which limits the generalizability to other regions and resident populations with different baseline training levels.

Future efforts include assessing the long-term clinical impact of teaching breast health using the JTS model. Chart review, focusing on increases in appropriate ordering of screening mammograms, documentation of family history in high-risk patients, and discussions regarding appropriate screening discontinuation would allow us to evaluate the lasting impact the JTS curriculum had on resident clinical practice. By expanding the use of JTS to a broader range of medical topics, future research can examine if this method leads to higher long-term retention, critical thinking, and collaboration among residents as compared to traditional classroom methods. Future research should also utilize validated assessment instruments, linked pre- and postsession surveys, and control group comparisons to further evaluate the effectiveness of JTS.

## Appendices


Facilitator Guide.docxBreast Health Cases for Facilitators.docxBreast Health Cases for Learners.docxBreast Health Jigsaw Teaching Strategy.pptxPre- and Postsession Survey.docx
*All appendices are peer reviewed as integral parts of the Original Publication.*
