# Supplementary material for: Educating Internal Medicine Residents About Breast Health Using a Jigsaw Teaching Strategy: An Interactive Curriculum Module
Source: MedEdPORTAL. 2026 Nov 12;22:11652. doi: 10.15766/mep_2374-8265.11652 (PMC13755890; doi:10.15766/mep_2374-8265.11652)
Supplement: Supplementary file 1 [file mep_2374-8265.11652-s001.zip › B. Breast Health Cases for Facilitators.docx]

**Educating Internal Medicine Residents About Breast Health Using a Jigsaw Teaching Strategy: An Interactive Curriculum Module**

**Timeline**

Pre-survey and introduction: 5 minutes

Breakout into Round 1 Groups: 5 minutes

Breakout into Round 2 Groups: 20 minutes

Group discussion/Wrap-up: 5 minutes

Post-survey: 2 minutes

**Breast Health Patient Scenario #1**

**Objectives**:

1. Identify first line imaging choices for young low risk patients with breast symptoms

**HPI:** 18-year-old female presents with complaints of a breast mass in her left breast. She’s noticed it for the past 6 months. She checks on the mass occasionally when she showers, and feels like sometimes the mass is larger and sometimes it disappears. There’s no associated nipple discharge or fevers/chills. Occasionally she feels like the breast is tender, but not always.

**Gyn history**: Menarche at age 14 with a menstrual cycle every 28 days lasting for 6 days

**PMHx**: PCOS

**PSx:** none

**Medications**: metformin

**Family Hx**: Mom: hypertension

**Social Hx:** Lives at home with her parents and commutes to community college daily. Not sexually active.

**Breast Exam**:

·	Consistency of the breast: lumpy bumpy breast consistent with normal exam

·	No obvious breast skin lesions

·	No cervical, supraclavicular or axillary lymphadenopathy

·	Left breast with a small area (0.5 x 0.5cm) with a rope-like texture in the outer quadrant

·	No nipple discharges

You bring her back to the clinic 2 weeks later and are now unable to palpate that breast lump.

**Assessment**: *18yo/F w/ PCOS coming in with a complaint of a new painless breast mass.*

**What do you suspect she has? What would you order next for this patient?**

*This patient likely has fibrocystic breast changes where she’ll feel the breast changes/tenderness depending on her menstrual cycle. For confirmation, you could order a breast ultrasound.*

*According to the American College of Radiology (ACR) guidelines: women under 30 years old with a palpable breast mass warrants a breast US, and deems mammogram/tomosynthesis imaging usually not appropriate.*

*If the mass isn’t visualized or if they receive a score of BIRADS 4 and over, they will need further imaging and workup including a mammogram and US guided FNA/CNB.*

**A biopsy is warranted for the suspicious mass seen in the US even if the mass is not seen on the mammogram.*

***Preceptors:*** *please go over BIRADS scoring with the residents*

***BI-RADS 4 or higher warrant additional breast imaging such as a diagnostic mammogram or core biopsy. For scores less than BI-RADS 4, no further additional imaging is necessary.*

*References:*

1. [*Expert Panel on Breast Imaging, Klein KA, Kocher M, et al. ACR Appropriateness Criteria® Palpable Breast Masses: 2022 Update. J Am Coll Radiol 2023; 20:S146.*](https://www.uptodate.com/contents/diagnostic-evaluation-of-suspected-breast-cancer/abstract/1)
2. *US Preventive Services Task Force. Screening for Breast Cancer: US Preventive Services Task Force Recommendation Statement. JAMA. 2024;331(22):1918–1930. doi:10.1001/jama.2024.5534*

**Breast Health Patient Scenario #2**

**Objectives:**

1. Risk stratify patients who are at a high risk for breast and other related cancers.

**HPI:** 25-year-old female presents for an annual exam. ROS is unremarkable and has no complaints. She mentions that she is concerned about her family history of cancer.

**Gyn History**: Menarche at age 15; menstrual cycle every 28 days, lasting for 4 days.

**Past Medical History:** Nothing

**Past Surgical History:** Nothing

**Family Hx:**

·	Maternal grandmother was diagnosed with breast cancer at age 65, treated

·	Grandfather passed away from colon cancer at 85

·	Pancreatic cancer in paternal uncle

**Medications:** none

**Social Hx:** Lives with a roommate and is studying to become a physician assistant; Occasional alcohol and marijuana use weekly; Sexually active with one male

**Breast Exam:**

·	Consistency of the breast: lump bumpy consistent with normal exam

·	No obvious breast skin lesions

·	No cervical, supraclavicular or axillary lymphadenopathy

·	No lumps found on exam

·	No nipple discharge

**Assessment:** *25-year-old female with a significant familial history of breast cancer in a first degree relative presents for an annual exam.*

**What would you order? What are the next follow up steps?**

1. *Genetic screening for high risk breast cancer mutations, including BRCA1/BRCA2, TP53, PTEN, etc.*
   a. *A patient with a familial history of breast cancer in a first degree relative before the age of 50, should trigger a provider to complete a familial risk assessment tool that determines the risk of having an inherited mutation. Women with a positive result on the risk assessment tool should receive genetic counseling.*
      i. *Tyrer-Cyzick Calculator - Based on familial and personal risk, displays lifetime risk score for developing breast cancer.*
         1. *Includes: age, height & weight, breast density, history & results of breast biopsies, personal history of breast or ovarian cancer, family history of breast or ovarian cancer, history of hormone use, Ashkenazi Jewish heritage, age of menstrual period, age of first pregnancy, age at menopause, & age of cancer diagnosis.*
      ii. *Other screening tools include the Ontario Family History Assessment or the Manchester Scoring System* *if concerned for BRCA mutations.*
      iii. *Depending on the score, a recommendation can be made for genetic counseling and further testing.*
   b. *Important to recognize other genetic syndromes and mutations to screen for when counseling patients with a familial history of cancer.*
      i. *Lynch Syndrome - associated with cancers of the uterus, ovary, stomach, pancreas, kidney, bladder and ureters (the tubes connecting the kidneys to the bladder), prostate, small bowel, biliary tract, brain, and skin (such as squamous cell carcinoma).*
         1. *Screening: The Amsterdam criteria, assess risk based on familial cancer history. The Bethesda criteria, assess risk of Lynch syndrome in people diagnosed with colon cancer.*
      ii. *Li-Fraumeni Syndrome - Patients with a diverse spectrum of cancers in their familial history may trigger screening*
          1. *Screening for this syndrome is still under investigation.*
   c. ***Guidelines for decreasing breast cancer risk in genetic mutation carriers:*** *Interventions include earlier and more frequent cancer screenings, including mammography, ultrasound, clinical breast exams and MRI.*
      i. *Imaging: A new study of BRCA1 and BRCA1 mutation carriers demonstrated the importance of ANNUAL MRI and mammogram starting at age 30.*
      ii. *Chemopreventive agents: Clinical trials that include selective estrogen receptor modulators and aromatase inhibitors that showed significant reduction in invasive breast cancer (none were studied among genetic mutation carriers).*
      iii. *Prophylactic surgeries: Prophylactic mastectomy and salpingo-oophorectomy were associated with decreased breast and ovarian cancer risk, while may be associated with other surgical side effects.*

*The American Cancer Society states that the following women should undergo strict surveillance as stated above. Women with:*

- *A lifetime risk of breast cancer of 20% or greater, according to risk assessment tools*
- *A known BRCA mutation after genetic testing*
- *A first-degree relative with a BRCA mutation, and have not yet tested*
- *A history of radiation therapy to the chest before they were 30 years old*
- *Li-Fraumeni syndrome, Cowden syndrome, or Bannayan-Riley-Ruvalcaba syndrome, or have first-degree relatives with one of these syndromes*

*There’s not enough evidence to make a recommendation for or against yearly MRI screening for women who have a higher lifetime risk based on certain factors, such as:*

- *Having a personal history of breast cancer,* [*ductal carcinoma in situ (DCIS)*](https://www.cancer.org/cancer/types/breast-cancer/about/types-of-breast-cancer/dcis.html)*,* [*lobular carcinoma in situ (LCIS)*](https://www.cancer.org/cancer/types/breast-cancer/non-cancerous-breast-conditions/lobular-carcinoma-in-situ.html)*,* [*atypical ductal hyperplasia (ADH), or atypical lobular hyperplasia (ALH)*](https://www.cancer.org/cancer/types/breast-cancer/non-cancerous-breast-conditions/hyperplasia-of-the-breast-ductal-or-lobular.html)
- *Having “extremely” or “heterogeneously”* [*dense breasts*](https://www.cancer.org/cancer/types/breast-cancer/screening-tests-and-early-detection/mammograms/breast-density-and-your-mammogram-report.html) *as seen on a mammogram*

**References:**

1. *Asdahl PH, Ojha RP, Hasle H. Cancer Screening in Li-Fraumeni Syndrome. JAMA Oncol. 2017;3(12):1645–1646. doi:10.1001/jamaoncol.2017.2459*
2. *“BRCA1 and BRCA2 (PDQ®) - NCI.” Www.cancer.gov, 3 Mar. 2023, www.cancer.gov/publications/pdq/information-summaries/genetics/brca-genes-hp-pdq#_3492.*
3. *US Preventive Services Task Force. Screening for Breast Cancer: US Preventive Services Task Force Recommendation Statement. JAMA. 2024;331(22):1918–1930. doi:10.1001/jama.2024.5534*
4. *Saslow D, Boetes C, Burke W, et al. American Cancer Society guidelines for breast screening with MRI as an adjunct to mammography. CA Cancer J Clin. 2007 Mar-Apr;57(2):75-89.*
5. Hornbuckle K, Fritz CDL. What Is Lynch Syndrome? JAMA. 2024;332(2):178. doi:10.1001/jama.2024.8552

**Breast Health Patient Scenario #3**

**Objectives:**

1. Describe the different breast cancer screening guideline recommendations from USPSTF, ACOG, ACS, ACR
2. Interpret mammogram reports (ie: dense breasts, breast calcifications)

**HPI:** 40-year-old female presents for comprehensive physical exam.

**Gyn history**: menarche at aged 13; menstrual cycles every 27 days, lasting 4 days

**Past medical history:** GERD
**Past surgical history:** 2 C-sections, one at age 28 and one at age 32

**Medications:** Omeprazole daily

**Allergies:** Shellfish

**Family History:** Mother – Hypertension; Father – Deceased from a cardiac event

**Social History:** Lives with her partner and 2 children; Does not smoke tobacco, drink alcohol or participate in recreational drug use; Uses condoms for contraception

**Healthcare maintenance:** Pap UTD; No previous breast imaging; Vaccinations UTD.

**Breast exam:**

·	Consistency of the breast: lumpy bumpy consistent with regular exam

·	No obvious breast skin lesions

·	No cervical, supraclavicular or axillary lymphadenopathy

·	No nipple discharges

·	No lesions noted on breasts bilaterally

**Assessment:**

*40-year-old female with a past medical history of GERD presenting for physical exam.*

**What would you order for this patient? Based on her mammogram, she has very dense breasts- what further imaging would be recommended for her?**

1. *This patient is recommended to begin screening for breast cancer.*

2. *Screening mammograms determine if a woman has dense breasts NOT clinical breast exams. Dense breasts are associated with an increased risk of breast cancer such that mammograms may miss lesions. More research is needed on best additional screening modalities for women with dense breasts, whether that is by adding an tomosynthesis, ultrasound, an MRI, or something else entirely. USPSTF guidelines (stated below) conclude that there is insufficient evidence to assess if adding ultrasonography or MRI in women with dense breasts is beneficial or harmful for screening.*

*BI-RADS classifies breast density into four categories:*

1. *Entirely* [*fatty breast tissue*](https://www.cancer.gov/Common/PopUps/popDefinition.aspx?id=CDR0000044759&version=Patient&language=en)*: There is almost all fatty breast tissue. It is found in about 10% of women.*
2. [*Scattered fibroglandular breast tissue*](https://www.cancer.gov/Common/PopUps/popDefinition.aspx?id=CDR0000784772&version=Patient&language=en)*: There is mostly fatty tissue with some areas of dense glandular and fibrous connective tissue. It is found in about 40% of women.*
3. [*Heterogeneously dense breast tissue*](https://www.cancer.gov/Common/PopUps/popDefinition.aspx?id=CDR0000784774&version=Patient&language=en)*: There are many areas of dense glandular and fibrous connective tissue, with some areas of fatty tissue. It is found in about 40% of women.*
4. [*Extremely dense breast tissue*](https://www.cancer.gov/Common/PopUps/popDefinition.aspx?id=CDR0000784776&version=Patient&language=en)*: There is almost all dense glandular and fibrous connective tissue. It is found in about 10% of women.*
   a. *Although USPSTF states that there is not yet enough evidence to recommend for or against additional screening with breast ultrasound or MRI -- common practice is to offer breast ultrasound if diagnosed with extremely dense breast tissue*

**References:**

1. *Lee SC, Pirikahu S, Fritschi L, Boyle T, Schultz C, Wylie E, Stone J. The association between breast arterial calcification and atherosclerotic cardiovascular disease in an Australian population-based breast cancer case-control study. Radiol Med. 2023 Apr;128(4):426-433. doi: 10.1007/s11547-023-01611-y. Epub 2023 Mar 6. PMID: 36877422; PMCID: PMC10119204.*
2. *US Preventive Services Task Force. Screening for Breast Cancer: US Preventive Services Task Force Recommendation Statement. JAMA. 2024;331(22):1918–1930. doi:10.1001/jama.2024.5534*

**Breast Health Patient Scenario #4**

**Objective:**

1. To identify when to discuss risk vs benefit of breast cancer screening in older patients
2. Discuss when to stop screening for breast cancer.

**HPI:** 74-year-old Caucasian female presents as a new patient visit for an annual physical exam. She feels well and has been attending her tennis class twice a week with only some occasional knee aches and pains, which Tylenol helps relieve. She heard about a friend being diagnosed with breast cancer at 75 years old and was worried that she’s missed mammograms for the last 3 years since her last primary care doctor retired and wants a referral for a mammogram.

**Past gyn history**: menstrual period started at 14 years old; menopause at 51 years old; 2 natural births (at aged 29 and 34). No previous breast biopsies. Never used hormonal therapy.

**Past medical history:** osteoarthritis of knees, HTN, HLD, pre-diabetes
**Past surgical history:** none

**Medications:** Atorvastatin 40mg once a day, amlodipine 10mg once a day, tylenol prn

**Allergies:** NKDA

**Family History:** Maternal grandmother – breast cancer at 60 years old; Father – HTN

**Social History:** Lives with her partner; Does not smoke tobacco, drink alcohol or participate in recreational drug use

**Healthcare maintenance:** Pap normal 3 years ago; last mammogram 3 years ago was normal

**Breast exam:**

·	Consistency of the breast: lumpy breast exam consistent with normal exam

·	No obvious breast skin lesions

·	No cervical, supraclavicular or axillary lymphadenopathy

·	No nipple discharges

·	No lesions noted on breasts bilaterally

**Assessment:** *74-year-old Caucasian female w/ PMHx HTN, HLD, OA, pre-DM here for annual visit to discuss necessity of future mammograms.*

**How would you advise this patient about when to stop screening? Are there any assessment tools that can be used to help guide your decision?**

*USPSTF guidelines state that above 75 years old, there should be a discussion between physician and patient with goals of care and whether a breast cancer diagnosis would be acted on based on functional status.*

*To help with that assessment, there are risk factor models to help assess pt’s risk and help guide the discussion: Gail Model: estimates absolute risk of developing invasive breast cancer during the next 5-year period and up to age 90 (lifetime risk) based on:*

- *Age*
- *Age at the start of menstruation*
- *Age at first live birth of a child*
- *Number of first-degree relative (mother, sisters, daughters) with breast cancer*
- *Number of previous breast biopsies (whether positive or negative)*
- *Presence of atypical hyperplasia in a biopsy*

*Optional: Breast cancer risk assessment calculator (the GAIL model). The Breast Cancer Risk Assessment Tool. Accessed March 10, 2026.* [*https://bcrisktool.cancer.gov/calculator.html*](https://bcrisktool.cancer.gov/calculator.html)

- *Clicking on the link will bring you to the Gail Model calculator to demonstrate how you would put in a patient’s information to calculate her risk*

*This tool cannot accurately calculate risk for women with a medical history of breast cancer, DCIS or LCIS.*

*Because her Gail risk score is >3%, she can be offered chemoprevention (SERMs, Aromatase Inhibitors) if no contraindications (ie: hx of VTE/PE, stroke, etc).*

- *Tamoxifen is used for chemoprophylaxis primarily in premenopausal women and in postmenopausal women who have had a hysterectomy; raloxifene or AIs are used in postmenopausal women.*

*Her past mammograms have stated that she has Tyrer-Cuzick Lifetime Risk: 4.2% (will be reported on mammogram but it’s a lifetime risk assessment and may not be actionable). If you plug in her information to the Tyrer-Cuzick calculator, this is what it would show:*

*References:*

1. *Breast cancer risk assessment calculator (the GAIL model). The Breast Cancer Risk Assessment Tool. Accessed March 10, 2026.* [*https://bcrisktool.cancer.gov/calculator.html*](https://bcrisktool.cancer.gov/calculator.html)
2. *US Preventive Services Task Force. Screening for Breast Cancer: US Preventive Services Task Force Recommendation Statement. JAMA. 2024;331(22):1918–1930. doi:10.1001/jama.2024.5534*
