# Supplementary material for: Educating Internal Medicine Residents About Breast Health Using a Jigsaw Teaching Strategy: An Interactive Curriculum Module
Source: MedEdPORTAL. 2026 Nov 12;22:11652. doi: 10.15766/mep_2374-8265.11652 (PMC13755890; doi:10.15766/mep_2374-8265.11652)
Supplement: Supplementary file 1 [file mep_2374-8265.11652-s001.zip › C. Breast Health Cases for Learners.docx]

**Breast Health Patient Scenario #1**

**Objectives**:

1. Identify first line imaging choices for young low risk patients with breast symptoms

**HPI:** 18-year-old female presents with complaints of a breast mass in her left breast. She’s noticed it for the past 6 months. She checks on the mass occasionally when she showers, and feels like sometimes the mass is larger and sometimes it disappears. There’s no associated nipple discharge or fevers/chills. Occasionally she feels like the breast is tender, but not always.

**Gyn history**: Menarche at age 14 with a menstrual cycle every 28 days lasting for 6 days. LMP 9/30/2024

**PMHx**: PCOS

**PSx:** none

**Medications**: metformin

**Family Hx**: Mom: hypertension

**Social Hx:** Lives at home with her parents and commutes to community college daily. Not sexually active.

**Breast Exam**:

·	Consistency of the breast: lumpy bumpy breast consistent with normal exam

·	No obvious breast skin lesions

·	No cervical, supraclavicular or axillary lymphadenopathy

·	Left breast with a small area (0.5 x 0.5cm) with a rope-like texture in the outer quadrant

·	No nipple discharges

You bring her back to the clinic 2 weeks later and are now unable to palpate that breast lump.

**Assessment**:

**What do you suspect she has? What would you order next for this patient?**

**Breast Health Patient Scenario #2**

**Objectives:**

1. Risk stratify patients who are at a high risk for breast and other related cancers.

**HPI:** 25-year-old female presents for an annual exam. ROS is unremarkable and has no complaints. She mentions that she is concerned about her family history of cancer.

**Gyn History**: Menarche at age 15; menstrual cycle every 28 days, lasting for 4 days.

**Past Medical History:** Nothing

**Past Surgical History:** Nothing

**Family Hx:**

·	Maternal grandmother was diagnosed with breast cancer at age 65, treated

·	Grandfather passed away from colon cancer at 85

·	Pancreatic cancer in paternal uncle

**Medications:** none

**Social Hx:** Lives with a roommate and is studying to become a physician assistant; Occasional alcohol and marijuana use weekly; Sexually active with one male

**Breast Exam:**

·	Consistency of the breast: lump bumpy consistent with normal exam

·	No obvious breast skin lesions

·	No cervical, supraclavicular or axillary lymphadenopathy

·	No lumps found on exam

·	No nipple discharge

**Assessment:**

**What would you order? What are the next follow up steps?**

**Breast Health Patient Scenario #3**

**Objectives:**

1. Describe the different breast cancer screening guideline recommendations from USPSTF, ACOG, ACS, ACR
2. Interpret mammogram reports (ie: dense breasts, breast calcifications)

**HPI:** 40-year-old female presents for comprehensive physical exam.

**Gyn history**: menarche at aged 13; menstrual cycles every 27 days, lasting 4 days

**Past medical history:** GERD
**Past surgical history:** 2 C-sections, one at age 28 and one at age 32

**Medications:** Omeprazole daily

**Allergies:** Shellfish

**Family History:** Mother – Hypertension; Father – Deceased from a cardiac event

**Social History:** Lives with her partner and 2 children; Does not smoke tobacco, drink alcohol or participate in recreational drug use; Uses condoms for contraception

**Healthcare maintenance:** Pap UTD; No previous breast imaging; Vaccinations UTD.

**Breast exam:**

·	Consistency of the breast: lumpy bumpy consistent with regular exam

·	No obvious breast skin lesions

·	No cervical, supraclavicular or axillary lymphadenopathy

·	No nipple discharges

·	No lesions noted on breasts bilaterally

**Assessment:**

**What would you order for this patient?**

**Based on her mammogram, she has very dense breasts- what further imaging would be recommended for her?**

*‌*

**Breast Health Patient Scenario #4**

**Objective:**

1. To identify when to discuss risk vs benefit of breast cancer screening in older patients
2. Discuss when to stop screening for breast cancer.

**HPI:** 74-year-old Caucasian female presents as a new patient visit for an annual physical exam. She feels well and has been attending her tennis class twice a week with only some occasional knee aches and pains, which Tylenol helps relieve. She heard about a friend being diagnosed with breast cancer at 75 years old and was worried that she’s missed mammograms for the last 3 years since her last primary care doctor retired and wants a referral for a mammogram.

**Past gyn history**: menstrual period started at 14 years old; menopause at 51 years old; 2 natural births (at aged 29 and 34). No previous breast biopsies. Never used hormonal therapy.

**Past medical history:** osteoarthritis of knees, HTN, HLD, pre-diabetes
**Past surgical history:** none

**Medications:** Atorvastatin 40mg once a day, amlodipine 10mg once a day, tylenol prn

**Allergies:** NKDA

**Family History:** Maternal grandmother – breast cancer at 60 years old; Father – HTN

**Social History:** Lives with her partner; Does not smoke tobacco, drink alcohol or participate in recreational drug use

**Healthcare maintenance:** Pap normal 3 years ago; last mammogram 3 years ago was normal

**Breast exam:**

·	Consistency of the breast: lumpy breast exam consistent with normal exam

·	No obvious breast skin lesions

·	No cervical, supraclavicular or axillary lymphadenopathy

·	No nipple discharges

·	No lesions noted on breasts bilaterally

**Assessment:**

**How would you advise this patient about when to stop screening?**

**Are there any assessment tools that can be used to help guide your decision?**
