# Supplementary material for: Educating Internal Medicine Residents About Breast Health Using a Jigsaw Teaching Strategy: An Interactive Curriculum Module
Source: MedEdPORTAL. 2026 Nov 12;22:11652. doi: 10.15766/mep_2374-8265.11652 (PMC13755890; doi:10.15766/mep_2374-8265.11652)
Supplement: Supplementary file 1 [file mep_2374-8265.11652-s001.zip › E. Pre- and Postsession Survey.docx]

Presession Evaluation

Please complete the following survey on your understanding of women's health education. Your responses are confidential and will not be used in your evaluation.

* Indicates required question

1. What is your current year of training?*

- PGY 1
- PGY 2
- PGY 3

2. What is your gender? *

- Female
- Male
- Prefer not to say

3. How comfortable are you with the current breast cancer screening guidelines? *

1	2	3	4	5

Very Uncomfortable	Very Comfortable

4. Are you familiar with risk factor calculators that can be used to guide breast cancer screening? *

- Yes
- No

5. Do you know how to interpret BIRADS results? *

- Yes
- No

6. Do you know what breast imaging should be ordered for women under 30 years old with a new breast mass? *

- Yes
- No

7. How comfortable are you in doing a risk assessment for patients at higher than average risk for breast cancer? *

1	2	3	4	5

Very Uncomfortable	Very Comfortable

**Postsession Evaluation**

Please complete the following survey on your experience with today’s clinical skills session. Your responses are confidential and will not be used in your evaluation.

* Indicates required question

1. What is your current year of training? *

- PGY 1
- PGY 2
- PGY 3

2. What is your gender? *

- Female
- Male
- Prefer not to say

3. At what age should you start breast cancer screening according to USPSTF guidelines? *

_____________________________________________________________________________________

4. How comfortable are you with the current breast cancer screening guidelines? *

1	2	3	4	5

Very Uncomfortable	Very Comfortable

5. After this session, how comfortable are you in doing a risk assessment for patients at higher than average risk for breast cancer? *

1	2	3	4	5

Very Uncomfortable	Very Comfortable

6. What is the first-line imaging modality recommended for a 30-year-old female patient coming into the clinic with a breast mass? *

- Breast ultrasound
- Ultrasound guided biopsy
- Mammogram
- Breast MRI

7. Which of the following assessment tools can be used to assess lifetime breast cancer risk in a high-risk patient? *

- Gail Model
- Tyrer Cuzick Score
- Manchester Scoring System

8. Do you prefer a virtual format or an in-person format for this kind of lecture? *

- Virtual
- In-person
- No preference

9. What concepts are still confusing?

_____________________________________________________________________________________

_____________________________________________________________________________________

10. General Comments or Feedback

_____________________________________________________________________________________

_____________________________________________________________________________________

**ANSWER KEY**:

3. Age 40

6. Breast ultrasound

7. Tyrer Cuzick Score
