# Supplementary material for: Educating Internal Medicine Residents About Breast Health Using a Jigsaw Teaching Strategy: An Interactive Curriculum Module
Source: MedEdPORTAL. 2026 Nov 12;22:11652. doi: 10.15766/mep_2374-8265.11652 (PMC13755890; doi:10.15766/mep_2374-8265.11652)
Supplement: Supplementary file 1 [file mep_2374-8265.11652-s001.zip › A. Facilitator Guide.docx]

Appendix A: Facilitator guide

Here is a suggested timeline and steps to plan and implement the JTS for Breast Health education.

2-3 months before the session:

- Download and review all the materials - ESR, facilitator version of cases, participant version of cases, pre and post survey questions, powerpoint
- Set a date for the educational session and decide on format, in-person or synchronous virtual
  - If conducting this session in-person, select a room that would allow for multiple small group discussions. The ideal size of a small group is 6-8 learners per group in this format.
  - If conducting this session virtually, set up a ZOOM meeting with a small group break out room feature. The ideal size of a small group can be 4-6 learners per group in this format.
- Recruit faculty for lead facilitator and small group facilitator. The lead facilitator can also participate as a small group facilitator.

1-2 weeks before the session:

- Print sufficient copies of the facilitator version of the cases and resident version of the cases if teaching in-person. Email the resident version of the cases to residents if teaching virtually.
- Plan 4 small groups for JTS, both initial small groups and after rearranging the learners into their second groups.
- Update the powerpoint slides with the small group assignments..

At the teaching session:

- Lead facilitator to introduce the session, goals, learning objectives using the powerpoint slides. (5 minutes)
- Share the small group arrangement (round 1 and round 2). If virtual, create breakout groups. If in person, divide the room into small groups.
- Small group facilitators will facilitate the discussion during round 1 using the assigned cases. (5 minutes)
- Lead facilitator to reshuffle groups for the second round of discussion.
- Small group facilitators will facilitate discussion during round 2 for the cases. (20 minutes)
- The lead facilitator will conclude the session using the powerpoint slides to highlight the major take home points. (5 minutes)
